# Localized Plasticity in the Streamlined Genomes of Vinyl Chloride Respiring *Dehalococcoides*

**DOI:** 10.1371/journal.pgen.1000714

**Published:** 2009-11-06

**Authors:** Paul J. McMurdie, Sebastian F. Behrens, Jochen A. Müller, Jonathan Göke, Kirsti M. Ritalahti, Ryan Wagner, Eugene Goltsman, Alla Lapidus, Susan Holmes, Frank E. Löffler, Alfred M. Spormann

**Affiliations:** 1 Departments of Civil and Environmental Engineering and of Chemical Engineering, Stanford University, Stanford, California, United States of America; 2 Department of Biology, Morgan State University, Baltimore, Maryland, United States of America; 3 Department of Civil and Environmental Engineering, Georgia Institute of Technology, Atlanta, Georgia, United States of America; 4 Joint Genome Institute, Walnut Creek, California, United States of America; 5 Department of Statistics, Stanford University, Stanford, California, United States of America; Universidad de Sevilla, Spain

## Abstract

Vinyl chloride (VC) is a human carcinogen and widespread priority pollutant. Here we report the first, to our knowledge, complete genome sequences of microorganisms able to respire VC, *Dehalococcoides* sp. strains VS and BAV1. Notably, the respective VC reductase encoding genes, *vcrAB* and *bvcAB*, were found embedded in distinct genomic islands (GEIs) with different predicted integration sites, suggesting that these genes were acquired horizontally and independently by distinct mechanisms. A comparative analysis that included two previously sequenced *Dehalococcoides* genomes revealed a contextually conserved core that is interrupted by two high plasticity regions (HPRs) near the Ori. These HPRs contain the majority of GEIs and strain-specific genes identified in the four *Dehalococcoides* genomes, an elevated number of repeated elements including insertion sequences (IS), as well as 91 of 96 *rdhAB*, genes that putatively encode terminal reductases in organohalide respiration. Only three core *rdhA* orthologous groups were identified, and only one of these groups is supported by synteny. The low number of core *rdhAB*, contrasted with the high *rdhAB* numbers per genome (up to 36 in strain VS), as well as their colocalization with GEIs and other signatures for horizontal transfer, suggests that niche adaptation via organohalide respiration is a fundamental ecological strategy in *Dehalococccoides*. This adaptation has been exacted through multiple mechanisms of recombination that are mainly confined within HPRs of an otherwise remarkably stable, syntenic, streamlined genome among the smallest of any free-living microorganism.

## Author Summary

*Dehalococcoides* are free-living sediment and subsurface bacteria with remarkably small, streamlined genomes and an unusual degree of niche specialization. These strictly anaerobic bacteria gain metabolic energy exclusively through a novel type of respiration that results in reductive elimination of chlorides from organochlorines, many of which are priority pollutants. In this article, we compare the first complete genome sequences of *Dehalococcoides* strains that grow via respiration of vinyl chloride (VC), a human carcinogen and abundant groundwater pollutant. Our work provides novel insights into *Dehalococcoides* chromosome organization and evolution, identifies specific positions in the chromosomes where new genes—like the genes responsible for growth on VC—are integrated, and generates clues how these dechlorinating bacteria adapt to anthropogenic contamination. This information sheds new light on *Dehalococcoides* biology and ecology, with implications for enhanced bioremediation to protect dwindling drinking water reservoirs.

## Introduction

Vinyl chloride (VC) – a proven human carcinogen [1] – and other chloroethenes, such as trichloroethene (TCE) and tetrachloroethene (PCE), are among the most frequently detected groundwater contaminants in the United States of America and other industrialized countries [2]. Some members of a deeply branching Chloroflexi subphylum, the *Dehalococcoides* (Dhc), exhibit the unique ability to completely reduce these chloroethenes to ethene via VC as intermediate [3], thereby mediating a critical step in bioremediation of contaminated aquifers and subsurface environments [2]. Dhc are strictly anaerobic microorganisms with a highly specialized catabolism that is apparently restricted to organohalide respiration with molecular hydrogen as electron donor [3]–[6]. Despite some successful exploitation of Dhc activity for bioremediation, exploration of Dhc biology has been limited due to slow growth (doubling times in the laboratory between 19 hours [3] and 57 hours [7]), low per-cell biomass, as well as the absence of techniques for genetic manipulation.

Organohalide respiration in Dhc is catalyzed by heterodimeric, membrane-bound enzymes of about 500 aa in length, known as ‘reductive dehalogenases’ (RDases). The catalytically active ‘A’ subunit is believed to be anchored to the outside of the cytoplasmic membrane by a small (∼100 aa) predicted integral membrane ‘B’ subunit [8]. Two Dhc genome sequences of isolates that cannot respire VC revealed many full-length non-identical reductive dehalogenase homologous genes (*rdhAB*) per genome; 17 in *D. ethenogenes* strain 195 [9] and 32 in Dhc strain CBDB1 [10]. These genome sequences revealed a bias in the location of *rdhAB* and associated genes toward the origin of replication (Ori) and the leading strand [9],[10]. Several of these *rdhAB* were also found to be located nearby or within integrated elements (IEs) [11] or have a highly unusual codon bias [12] indicating possible horizontal acquisition. Culture-based studies have shown that strains 195 and CBDB1 contribute to dechlorination of a variety of priority pollutants including polychlorinated ethenes [13], benzenes [14], phenols [15], dibenzo-p-dioxins [16], dibenzofurans, biphenyls, and naphthalenes [14]. Notably, strain CBDB1 does not respire chloroethenes [5], and strain 195 cannot respire VC, instead exhibiting a slow cometabolic VC reduction activity [17].

Efforts to isolate microorganisms that can couple growth with VC dechlorination have resulted in the isolation of Dhc strains VS [18] and BAV1 [19], both of which share highly similar (>99% identical) 16S rRNA gene sequences with strains 195 and CBDB1. The VC RDases responsible for catalyzing VC transformation to ethene have been identified biochemically in strain VS [20], and are encoded by *vcrAB* in strain VS [20] and *bvcAB* in strain BAV1 [21]. To further understand Dhc genome organization and the genetic adaptations that led to VC respiration, we determined the complete genome sequence of Dhc strains VS and BAV1. To our knowledge, these are the first genome sequences of microorganisms able to grow by reductive dehalogenation of VC, a critical step for bioremediation of sites impacted by chlorinated ethenes [1]. Comparison between these VC respiring Dhc strains revealed that the VC RDase genes, *vcrAB* and *bvcAB*, are each located on distinct genomic islands (GEIs) at disparate locations of their respective genome, suggesting that the genetic basis for VC reduction was horizontally acquired in both strains through independent events. We also show that the integration site for the *vcr*-GEI is not unique to this GEI nor to strain VS, but instead appears to be an integration site associated with many other strain-specific *rdhA* in strains VS, 195, and CBDB1.

Similar to comparative analyses between other closely related, free-living bacteria (for example, [22],[23]), this four-way comparison of Dhc strains reveals that many strain-specific genes occur in a limited number of continuous segments and GEIs. Unlike those genomes, however, the GEIs and other strain-specific segments of Dhc are further clustered mainly within two regions positionally analogous to the ‘less-structured regions’ that flank the Ori-macrodomain in *Escherichia coli* [24]. These High Plasticity Regions include most examples of interruptions to core synteny between the four strains, including genomic rearrangements, IS elements and other repeated elements, insertions and deletions, as well as *rdhAB*. These HPRs reflect rapid evolutionary dynamics toward a presumed respiratory-niche diversification that contrasts an otherwise remarkably small and stable genome that, at 1.3–1.5 Mbp, is among the smallest of known free-living microorganisms [25]. This compartmentalization of genome dynamics to catabolism-associated Ori-flanking HPRs indicates an unusual biological solution to the opposing evolutionary pressures for genome streamlining and respiratory diversification.

## Results/Discussion

### Features and Organization of Strains VS and BAV1 Genomes

**Figure 1:**
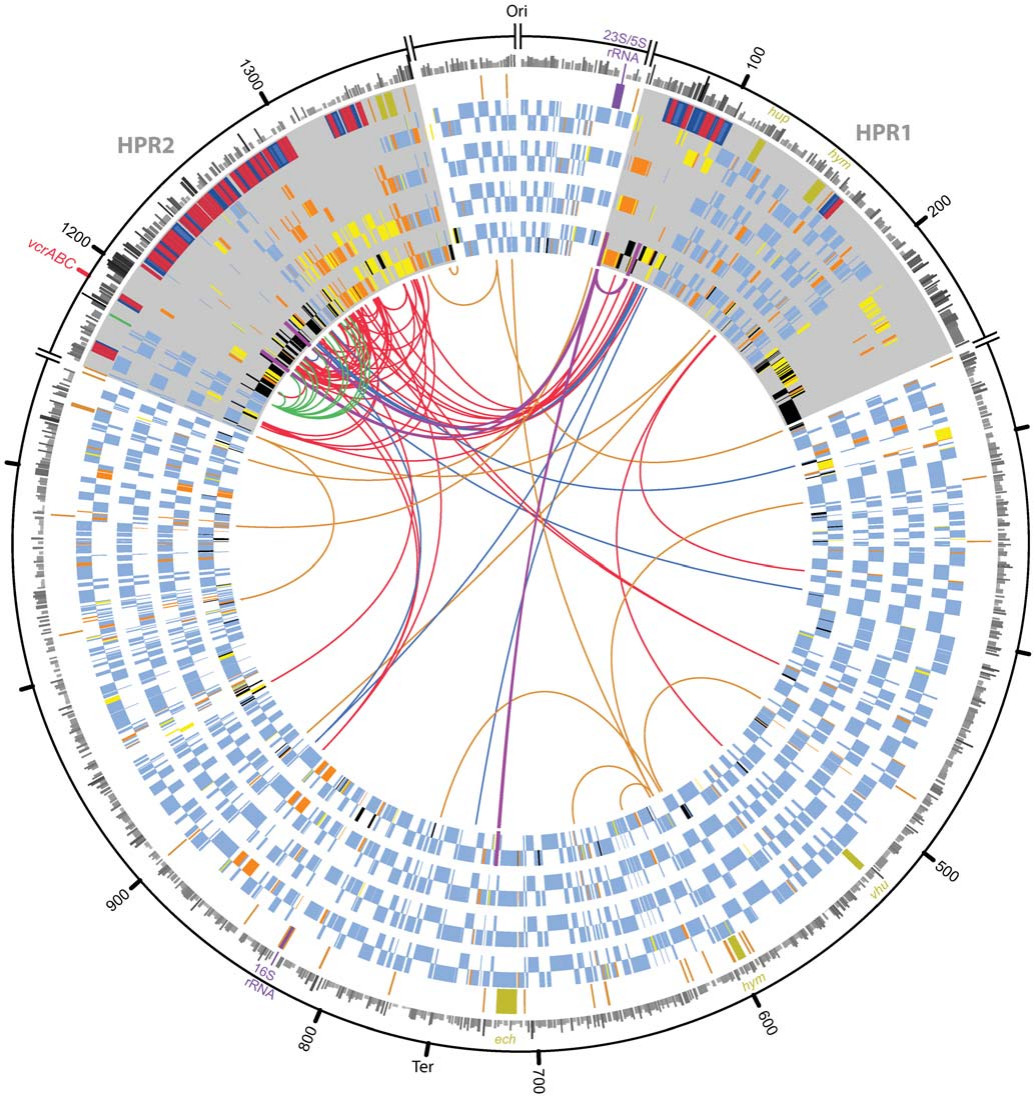
Circular map of *Dehalococcoides* (Dhc) genomes using strain VS as template. Genomic coordinates are shown in 100 kbp intervals beginning with the origin of replication. The rings depict from outside to inside: (Ring 1) Codon usage bias (CUB) [76] of each gene larger than 200 codons, shaded in greyscale according to their CUB value for emphasis, with black indicating highest CUB value. (Ring 2) Location of genes of special interest, shaded according to the following legend: (red) *rdhA* and *rdhB*, (blue) other *rdh*-associated, (dark yellow) hydrogenase, (violet) rRNA, (dark green) *ssrA*, (dark orange) tRNA. (Rings 3–6) Ortholog multiplicity of genes. Genes with orthologs in 3, 2, 1, or 0 of the other strains are shaded green, orange, yellow and black, respectively. Genes are drawn in Rings 3–5 only if orthologs to strain VS were detected in strain BAV1, strain 195, or strain CBDB1, respectively. Ring 6 shows the position and orientation of all predicted CDS in strain VS, shaded according to ortholog multiplicity. Also shown in Ring 3 is the location of IS elements (purple transecting bar). Genes in the outer or inner half of Rings 3–6 are in the forward or reverse direction, respectively. Grey wedge highlights are layered behind Rings 2–6 to indicate the positions of the high plasticity regions, HPR1 and HPR2, with small axis breaks also denoting these boundaries. Repeated elements overlapping any part of the genes of special interest in Ring 2 (excluding H2ase genes) are drawn as connected bezier ribbons, shaded according to the same legend, and with their radial span at each end indicating the length of the respective element.

**Figure 2:**
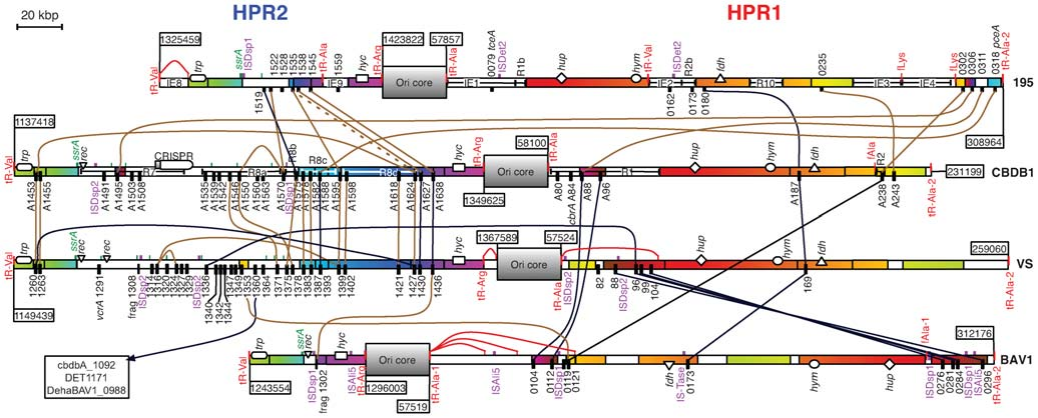
Scaled map of homologous in both HPR regions of each strain. All but two *rdhA* (DET0876, DehaBAV1_0847 – *bvcA*) are depicted here as black bottom-side tick marks. Large orthologous regions are shaded in the same color, in a gradient that indicates their relative orientation. Unfilled (white) boxes indicate strain-specific regions. Brown and black curves indicate less than 0.05 and 0.01 respective Jukes-Cantor distance for orthologous *rdhA*, or less than 0.10 and 0.01 Jukes-Cantor distance between *rdhA* paralogs, respectively (see Figure S7). Unless otherwise noted, a commutative property is implied for *rdhA* orthologs/paralogs that are not on adjacent strains in the diagram. Small top-side green ticks indicate the location of intergenic *ssrA* (tmRNA gene) repeated fragments. A fatter green tick mark indicates *ssrA*, iteslf. tRNA genes are indicated with red top-side tick marks and are accompanied with the abbreviated labels, “tR-Aaa.” Repeated fragments of tRNA genes are labeled ‘fAaa’ or connected to the presumed source gene with a red curve. A large black oval indicates the only CRISPR region detected in these genomes, in strain CBDB1 [10]. A triangle, oval, diamond, rectangle, and curved rectangle are used to indicate the location and size of the *fdh*, *hym*, *hup*, *hyc*, and *trp* operons, respectively. An upside-down triangle indicates a homologous recombinase-containing gene cluster apparently involved in *ssrA*-specific integration, and is present in VS, CBDB1, and BAV1 (see Figure S9). A wide middle box labeled “Ori core” represents approximately 130 kbp of synteny-supported, core genome that separates the two HPRs and includes the Ori. The chromosomal location of the edges of these regions are indicated with flags.

The genomes of strains VS and BAV1 are similar in total length (1413462 and 1341892 bp, respectively), % (G+C) (47.3% and 47.2%, respectively), as well as overall structure (Figure 1, Figure S1) to those of strains 195 and CBDB1 [9],[10]. There are 1029 orthologous groups of protein encoding genes (CDS) that are conserved across all four Dhc genomes, henceforth referred to as the core genome. The core genome accounts for 68 to 77% of a strain's genome (Table S1) and, generally speaking, these genes share the same order, orientation and genomic context (synteny) and encode the essential metabolic functions (Table S2) previously described for strains 195 and CBDB1 [9],[10]. GC-skew maps (Figure S2) identified a consistent predicted location for the origin and termini of replication of each Dhc strain and also revealed separate inversions in strains VS and BAV1 that are further supported by a corresponding disruption to gene synteny (Figure 1, Figure S1). Genome-level relationships were estimated by concatenation and multiple alignment of the core genome of each strain. The resulting phylogenetic tree is consistent with a previous 16S rRNA gene-based structure [26] (Figure S3). The core genomes of the two strains from the Pinellas phylogenetic subgroup, BAV1 and CBDB1, were found to be extremely similar with a median nt identity of core CDS >99%. By contrast, strain 195 (Cornell subgroup), strain VS (Victoria subgroup), and the Pinellas subgroup are separated by comparable Jukes-Cantor genetic distances. Disruptions to gene synteny occur predominately within two regions on either side of the Ori (Figure 1). These regions are variable in length (up to 200 kbp) and contain elevated occurrences of genomic islands (GEIs), insertion sequences (IS), other repeated elements, as well as apparent insertions, deletions and inversions. These regions account for less than one-fourth the cumulative length of the four genomes but contain 91 of 96 *rdhA*. We designated these two High Plasticity Regions, HPR1 and HPR2, and identified tRNA genes at their conserved boundaries (Figure 1, Figure 2).

### High-Plasticity Regions (HPRs)

HPR1 begins following tRNA-Ala-1 approximately 60 genes forward from the Ori and ends at tRNA-Ala-2 approximately 200 genes downstream (Figure 2). HPR1 of each strain contains examples of genomic rearrangements and GEIs that appear to have integrated at tRNA-Ala. Strain 195 also contains previously described GEIs that integrated at tRNA-Val as well as a repeated GEI that integrated at tRNA-Lys [9]. Recently repeated ISDsp2 and ISDsp1 or ISAli5 IS elements can be found in HPR1 of strains VS and BAV1, respectively. Additionally, two non-identical ISDet2 type IS elements are located in HPR1 of strain 195. Whole genome alignment, the order and orientation of orthologous genes (Figure S1), as well as GC-skew analysis (Figure S2) show that the majority of HPR1 in strain BAV1 was recently inverted, and also that at least one inversion has occurred in HPR1 of strain VS. These are the only inversions >40 kbp in length detected in the four strains. HPR1 includes a total of 30 *rdhA*, 7 of which are strain specific. This region also includes ∼100 kbp of core genes with occasionally interrupted order, many of which are believed to be involved in key catabolic functions, including the *hup* operon (encoding [NiFe-(Se)] uptake hydrogenase), the *hym* operon (encoding [Fe] hydrogenase), and the *fdh*-like operon that is strongly expressed in dechlorinating Dhc cultures [27]. Also within this region is the only conserved full-length *rdhA* with an orthology status that is also supported by synteny in all four strains (DehaBAV1_0173, cbdb_A187, DET0180, DhcVS169, Figure 2).

HPR2 begins with a ∼30 kbp region of atypically high similarity (>99% nt identity) between strain 195 and the Pinellas strains following a conserved set of three tRNA genes (tRNA-Leu,Arg,Val). A phylogram based on a multiple alignment of this region is incongruent with the phylogeny estimated for the rest of the genome (Figure S3, Figure S4), suggesting a xenologous displacement of this region in strain 195, sourced from a Pinellas strain. Probes for this region of strain 195 were among the only highly conserved oligonucleotides that failed to hybridize to genomic DNA from ANAS, a culture highly enriched in different Cornell strains [28],[29]. The dubious evolutionary history of this region and its proximity to *rdhAB* in HPR2 is especially interesting because it includes the biosynthesis operon for tryptophan, a significantly enriched [10] and positionally conserved [21] residue in RdhB sequences (Figure S5).

This ∼30 kbp region, potentially displaced in strain 195, is followed by a region with the highest density of *rdhA* in the genomes (discussed below). Following this, the terminal ∼40 kbp of HPR2 is a syntenic region shared between strains CBDB1 and VS, and includes six orthologous pairs of *rdhA* (Figure 2). The last 12.5 kbp of this region is also syntenic in strain 195, encompassing two *rdhA* orthologous triplets (DhcVS1430/cbdb_A1627/DET1538; DhcVS1436/cbdb_A1638/DET1545). The final *rdhA* gene of HPR2 has syntenic representatives in all four strains (DehaBAV1_1302, cbdb_A1638, DET1545, DhcVS1436), but this *rdhA* gene is present in BAV1 only as a N-terminal fragment that stops abruptly at the boundaries of an ISDsp1 type IS element. This genome truncation in strain BAV1 (CBDB1 equivalent locations 1174623–1326022, 151 kbp) accounts for its lower total number of *rdhA* and explains the shorter overall length of the BAV1 genome. Similarly, a deletion event appears to have taken place in the strain 195 genome between two *rdhA* (corresponding to DhcVS1399 and DhcVS1427), leaving behind the apparently chimeric *rdhA*, DET1535 (Figure 2, Figure S6).

### Reductive Dehalogenase Homologous Genes

**Figure 3:**
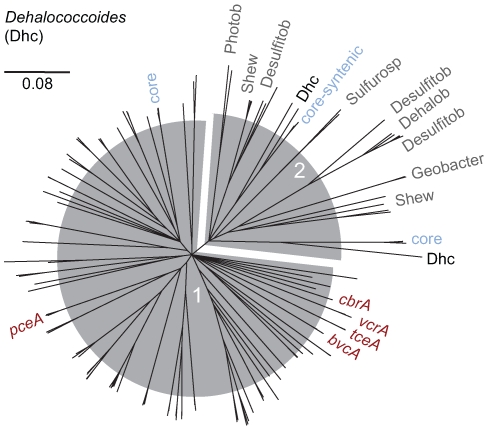
Phylogenetic overview of available RdhA sequences. Radial Neighbor-Joining consensus tree of the deduced amino acid sequence from all full-length *rdhA* available in public databases. Leaves represent RdhA derived from *Dehalococcoides* (Dhc) unless otherwise indicated. RdhA sequences from Dhc strain FL2 and Dhc-containing enrichment culture KB1 are included. Orthologous groups with a representative in all 4 presently sequenced Dhc strains (core) are labeled and shaded in light blue as in Figure 1. RdhA from non-Dhc microorganisms are labeled with the following abbreviations: Dehalob *Dehalobacter*.; Desulfitob *Desulfitobacterium*; Photobac *Photobacterium*; Sulfurosp *Sulfurospirillum*; Shew *Shewanella*. A vertical tree with comprehensive leaf labels is provided in Figure S7.

*rdhAB* and genes believed to be involved in assembly and maturation (*rdhF-I*) or regulation (*rdhC*, *D*, *R*) [10] comprise between 3.5 and 8.6% of these genomes by length. Strain VS contains 36 full-length *rdhA*, the most of any genome to date and the most unique (15 *rdhA*) among the four Dhc strains. While 32 of the 96 *rdhA* are unique to an individual strain, the remaining genes have at least one predicted Dhc ortholog. Most ortholog pairs are present in the same HPR and supported by local synteny (Figure 2). This emphasizes that many *rdhA* have been vertically inherited or horizontally acquired *en bloc* from another Dhc, consistent with a previous observation that many *rdhA* have a codon usage that is indistinguishable from the rest of the genome [12]. Furthermore, available *rdhA* cluster into two major phylogenetic clades, the largest of which (Cluster 1) contains only Dhc-derived *rdhA*. Cluster 1 also includes the VC RDase genes *bvcA* and *vcrA*, the TCE RDase gene *tceA*, the PCE RDase gene, *pceA* [13],[30], the chlorobenzene RDase gene, *cbrA* [31], and a total of 85 *rdhA*. Notably, Cluster 2 contains two of the Dhc core *rdhA* groups, in addition to all presently available non-Dhc *rdhA* (Figure 3, Figure S7). Though obscured somewhat by rearrangements and HGT, as many as 19 contemporary *rdhA* orthologous groups may have been present in the most recent common ancestor (Figure 2, Figure S8). Overall, HGT between Dhc strains, horizontal transfer from non-Dhc, interruption, and deletion all appear to contribute to the evolution and availability of *rdhA* in Dhc.

### Horizontal Transfer of Key RDase Genes

In strains VS and BAV1, the respective VC RDase-encoding operons are located on distinct GEIs (Figure S9). In BAV1, *bvcAB* is one of only two *rdh* operons outside of the HPRs, and it is embedded in a GEI that is flanked on either side by a tRNA-Arg and a 58 bp directly repeated fragment of its 3′ end. This GEI has an unusually low %(G+C) and also contains a putative integrase, DehaBAV1_0846 [12]. Similarly, *vcrAB* of strain VS is embedded within a low %(G+C) GEI in HPR2 that is flanked by *ssrA* – a single-copy essential gene [32] encoding transfer messenger RNA (tmRNA) [33] – and its 20 bp direct repeat (DR). This GEI (positions 1177831–1189175) also contains a putative integrase (DhcVS1282) gene as well as two other genes associated with recombination (DhcVS1283, DhcVS1286), consistent with the canonical description of *ssrA*-specific integration of GEIs described for other bacterial genomes [34]. This *vcrA*-containing, *ssrA* GEI (heretofore *vcr*-GEI) is followed immediately by another apparently *ssrA*-specific GEI, the first six genes of which (DhcVS1292-1298) comprise an integration-associated block that is homologous to the six genes immediately adjacent to *ssrA* in *vcr*-GEI (Figure S9). The distal boundary of this downstream tandem *ssrA* GEI is defined by a 19 bp *ssrA* DR, and the remaining genes have no detectable similarity to *vcr*-GEI. Syntenic homologs to these six genes are also adjacent to *ssrA* in CBDB1 (cbdb_A1480-A1486), and three of these are at the corresponding location in BAV1 as well (DehaBAV1_1297-1299). The *ssrA* DR is found a total of 11 times downstream of *ssrA* in strain VS, 5 times in strain CBDB1, and once in strain 195 (Figure 1, Figure 2), and is further supported by traces of sequence similarity in the 150 bp of intergenic sequence surrounding each DR. In total, 19 of the 32 strain-specific *rdhA* in these four genomes are colocated with these *ssrA* fragments in strains CBDB1 and VS (Figure 2). The relative abundance of *rdhA* in strains VS and CBDB1 is largely attributable to this segment of HPR2 (12 in CBDB1, 18 in VS), further implicating *ssrA*-specific integration as a key mechanism of novel *rdhA* acquisition in *Dehalococcoides*.

The tRNA-Ala gene also appears to be a recombination site for one or more strain-specific GEIs that contain *rdhA* and phage-associated genes. HPR1 of BAV1 contains four repeated 3′ fragments of tRNA-Ala-1 in close proximity to *rdhA*, and two were identified in HPR1 of CBDB1. One of the fragments in CBDB1 flanks a GEI (previously entitled *Region 1* [10]) that contains 4 *rdhA*, 2 of which are unique to CBDB1. In strain 195, both *IE1* (which includes *tceA*) and *IE9* also appear to have integrated at tRNA-Ala [9]. In strain VS, a 25 bp DR from tRNA-Ala-1 (DhcVS62) is accompanied by a highly similar truncated homolog (DhcVS107) of site-specific recombinase gene DhcVS63. This gene and tRNA-Ala DR flank an inversion within HPR1 (∼57500–103500) that includes 5 *rdhA* (1 unique, DhcVS82) and two ISDsp2 type IS elements. Similarly, two of the tRNA-Ala-1 repeated fragments in HPR1 of BAV1 form a pair of inverted repeats at the approximate boundary to the ∼100 kbp inversion in BAV1 (Figure 1). The observation that tRNA-Ala genes are found at each boundary of HPR1, as well as the downstream boundary of HPR2, suggests that tRNA-Ala is also a key recombination site for acquisition of novel genetic elements in *Dehalococcoides*.

In addition to the VC reductase genes, all other currently identified chloroethene reductive dehalogenase genes appear to occur on GEIs. Unlike the *pceAB*-containing catabolic transposon (Tn-Dha1) found in *Desulfitobacterium* [35],[36], we did not detect flanking IS elements or any other flanking repeats at the island containing *pceAB* in HPR1 of strain 195. We further observed that this island is homologous to sequence located within a larger region in HPR2 that is contextually conserved between CBDB1 and VS (Figure 2). This *pceAB* island in strain 195 therefore appears to be the result of an intragenomic rearrangement between the HPRs, or a horizontal acquisition from another closely related microorganism. DNA microarray hybridization failed to detect *pceA* in three other Cornell strains, namely the two strains in the ANAS enrichment [28],[29] as well as the recently described strain MB that nevertheless dechlorinates PCE [37]. Although highly similar (aa ID, 94.5% DhcVS1393, 93.7% cbdb_A1588), the function of the corresponding homologs to *pceA* in CBDB1 and VS is unclear, as neither CBDB1 [5] nor VS [38] appears capable of PCE respiration, and PceA from strain 195 was implicated as a bifunctional enzyme that also catalyzes the reductive dehalogenation of 2,3-dichlorophenol [30]. The typical codon usage bias of all three *pceA* homologs [12], as well as the conserved context of these genes in strains VS and CBDB1, suggests that these genes are not a recent addition to Dhc.

Despite the apparent specialization of Dhc to organohalide respiration, the question as to whether or not certain *rdhA* are essential for Dhc remains unanswered. This is partly due to the high number and diversity of Dhc *rdhA*, their rampant horizontal transfer, as well as the genetic intractability of Dhc [39]. The three orthologous groups of *rdhA* with members in all four strains are obvious candidates, but only one of these groups is supported by synteny – albeit local and inverted in BAV1. An additional 3 or more *rdhA* orthologous groups would have been classified as synteny-supported core genes, were it not for their deletion in HPR2 of strain BAV1, including the orthologous group containing DET1545 (Figure 2). Interestingly, DET1545 was found to be among just four *rdhA* strongly upregulated during the transition from exponential growth to late stationary phase in strain 195 with TCE as sole depleting electron acceptor [40]. Among the remaining 3 strongly upregulated *rdhA* upon entry to stationary phase, one is core supported by synteny (DET0180), and another (DET1535) is predicted to have been syntenic-core prior to the deletion of its ortholog in strain BAV1. The synteny-supported core *rdhA* in CBDB1, cbdbA187, was shown to be differentially upregulated during respiration of 1,2,3-trichlorobenzene (TCB) compared with 1,2,4-TCB, although transcripts of *cbrA* were most abundant in both conditions [39]. While the specific activity of the small subset of core *rdhAB* is unknown, their elucidation promises key insight into the biology of *Dehalococcoides*. The viability of strain BAV1, in spite of its loss of many otherwise conserved *rdhAB* in HPR2, is further evidence of a modular character of RdhAB activity. Taken in combination with the observation of high genome-wide similarity between strains CBDB1 and BAV1, as well as the phenotypic observation that BAV1 respires vinyl chloride – while CBDB1 does not – suggests that acquisition of a VC RDase operon is sufficient to confer VC respiration in Dhc.

### Concluding Remarks

We describe the emergence of a genomic structure in which *rdhA* and associated genes, as well as other strain-specific genes are concentrated predominantly within two HPRs near the Ori. Analogous compartmentalization of the chromosome into HPRs has been reported for diverse microbes such as *Streptomyces* [41], *Borrelia* [42], and *Haloquadratum* [43], and may facilitate adaptation while maintaining stability of the core genome [44],[45]. Dhc HPRs are at least partially explainable by their colocation with common integration sites for genetic elements [46], the most prominent of which are the structural RNA genes for tmRNA and certain tRNAs. A locally elevated concentration of GEIs, repeated elements, homologous genes, and other features associated with recombination may further contribute to any intrinsic instability of these regions. The chromosomal clustering of *rdhAB* into domain-like HPR structures may also facilitate the observed coexpression of multiple *rdhA* in response to limited or single electron acceptors [30],[39],[40],[47],[48] by allowing improved access of RNA polymerase to nearby exposed DNA – as has been proposed as a general explanation for short (≤16 kbp) and medium (∼100 kbp) range expression correlation patterns in some bacteria [44]. Any selective advantage by facilitated coexpression also contends with a higher likelihood of losing key *rdhAB* because they are in regions of high plasticity.

The elucidation of extremely small genomes of marine bacterioplankton like *Canditatus* Pelagibacter ubique [25] and *Prochlorococcus* [49] have led to the emergence of the genome streamlining hypothesis, attributed to purifying selection acting on very large and globally dispersed populations [50]. Our analysis provides insight into genome streamlining of a free-living microbial subphylum that is specialized for a fundamentally different lifestyle and environment, namely organohalide respiration in anoxic zones of the terrestrial subsurface. At 1371 predicted ORFs, the strain BAV1 genome contains just 17 and 71 additional ORFs than the respective genomes of *Cand*. P. ubique and OM43 strain HTCC2181 [51], the smallest of known free-living microorganisms. Unlike *Cand*. P. ubique however, BAV1 and the other Dhc strains do contain pseudogenes, transposons, and IS elements. Their modest % (G+C) and 10-fold larger median length of intergenic spacers also indicate that the selective pressures toward genome reduction have been somewhat different for Dhc, while still allowing a comparably small genome size. The presence of between 11 and 36 *rdhAB* per genome implies a respiratory flexibility that may allow Dhc to use a variety of halogenated compounds as terminal electron acceptors. With no apparent alternative energy conservation mechanism for Dhc other than organohalide respiration, niche specialization to low abundance, naturally occurring chloroorganic compounds seems to be the dominant ecological strategy of this unique group of microorganisms. The apparent emphasis of Dhc genome dynamics on *rdhAB* diversity – compartmentalized within specialized regions near the Ori – may enhance opportunistic adaptation to (new) respiratory niches while protecting a streamlined core genome that is highly adapted to life in the anoxic subsurface, as evidenced here by the recent site-specific acquisition of vinyl chloride reductase genes.

## Methods

### Cultivation of Strains and DNA Extraction

For isolation of genomic DNA, Dhc sp. strain VS and BAV1 were grown as previously described [6],[20]. Total DNA was extracted as described previously for strain VS by a freeze-thaw lysis, phenol-chloroform-isoamyl alcohol purification, and ethanol precipitation [52].

### Complete Genome Sequencing and Annotation

Whole genome shotgun sequencing (Sanger method) and automated assembly of the genomes of Dhc strains VS and BAV1 was performed at the U.S. Department of Energy's Joint Genome Institute (JGI) following their standard production sequencing protocols (http://www.jgi.doe.gov/sequencing/protocols/). A combination of randomly sheared libraries with inserts in the 3 kb and 8 kb size range was used for each strain, as well as some 40 kb inserts (fosmid) for strain BAV1. The initial assembly of each genome was constructed with the Paracel Genome Assembler (PGA), with manual correction of possible mis-assemblies by editing in Consed [53] and gap closure by primer walking. In strain VS, sequences originating from contaminant genomic DNA were excluded by their high %(G+C) bias and low coverage. Scaffolding of strain VS was further enhanced by comparison with the previously sequenced Dhc strains 195 and CBDB1 (CP000027 [9] and AJ965256 [10], respectively), and verified with primers designed by Projector2 [54] or the Primer3 [55] implementation in Geneious [56]. The complete circular consensus sequences of VS and BAV1 achieve 23X and 20X coverage, respectively (Table S1). They are available at JGI's Integrated Microbial Genomes (IMG) website (http://img.jgi.doe.gov [57]), with the respective taxon IDs 641380429 and 640427111 (Genbank CP000688).

Computational prediction of open reading frames (ORFs) utilized the output of GLIMMER [58] and CRITICA [59]. Identification of ORFs unnoticed during automated prediction was performed by manual inspection of intergenic regions using Artemis [60] and Geneious. Overlapping ORFs without a functional assignment, significant BLASTP hit [61], or orthologous annotation in the previously sequenced strains were discarded. Functional assignments were created using JGIs automated annotation pipeline, with extensive manual inspection supported by SMART [62] and KEGG database [63] analyses. Genes for tRNA and tmRNA were detected using tRNAscan-SE and ARAGORN, respectively [64],[65].

### Identification of Orthologs

We identified orthologous relationships between protein encoding genes of these four Dhc strains using the same five heuristic criteria described in the pair-wise comparison of Dhc strains 195 and CBDB1 [10], applied to an all-versus-all BLASTP search incorporating the genes from all four genomes [61]. A ‘greedy’ commutative property of ortholog pairs was assumed to create ortholog groups that also contain putative paralogs. Core CDS were defined as those in groups with at least one representative from each of the four genomes. Larger orthologous regions spanning multiple genes were identified at the nucleotide level through manual inspection of multiple whole genome alignments generated by Mauve version 2.2.0 [66] and Muscle version 3.6 [67] for refinement.

### Whole-Genome Strain Phylogeny and the Core Genome

For each genome the core CDS were uniformly ordered, oriented, and concatenated as a single nucleotide sequence. A multiple alignment was created for the resulting concatenation of core genes using Muave. Jukes-Cantor phylogenetic distances and a Neighbor-Joining consensus tree were calculated from this multiple alignment using Geneious.

### Identification of Repeated Elements and IS Elements

An exhaustive determination of repeated elements greater than or equal to 18 bp in length was performed on all four genomes using the repeat-match algorithm in MUMmer3 [68]. IS elements and IS-transposases were detected by BLAST and BLASTP searches based on those described for strains 195 and CBDB1 [9],[10], as well as manual inspection of the genomic context surrounding significant search hits to IS elements and transposases in the ISFinder database [69]. Recently repeated IS elements (perfect and nearly-perfect copies) were discovered by manual inspection of all repeated elements of an appropriate size (0.7–2.5 kbp) and comparison to the ISFinder database.

### RdhA Phylogeny

Tree reconstruction was performed with a total of 152 RdhA sequences, including the 96 RdhAs from the four complete Dhc genomes and 56 RdhAs from other Dhc strains and non-Dhc species available in public databases. Deduced amino acid sequences were aligned with Muscle 3.6. The phylogenetic trees were calculated using the neighbour joining and maximum likelihood methods of the MEGA 4.0 software package [70] as well as the PHYML [71] implementation in Geneious. The tree topology was tested by the application of a 20% positional conservatory filter. Stability of the tree topology was further refined by bootstrapping (1,000 replications). Only RdhAs ≥400 amino acids were included.

### Genome Graphics

Scaled circular representation of the four genomes (Figure 1), including Bezier ribbons indicating repeats, was created with the Perl-based package, *Circos* [72]. Custom algorithms written in R [73] were used to distill redundant repeat information, and combine multiple repeated elements into a representation with a single (arbitrary) source and many sinks. The stylized representation of orthologous regions and *rdhA* present in the HPRs (Figure 2) was drawn as vector graphics by manual overlay on the multiple whole genome alignment [66] of these regions. Nucleotide skews (Figure S2, top) were calculated according to the Oriloc [74] implementation in the seqinR package [75] of the R language for statistical computing [73].

## Supporting Information

Figure S1 Pairwise position of orthologs shared between *Dehalococcoides* (Dhc) strains 195 (blue), CBDB1 (dark green), and BAV1 (light green) with Dhc strain VS (red; horizontal axis). Unique genes are plotted along the axis (at zero) to which they belong. All genes are shaded according to the respective vertical axis genome. The position of *rdhA* are indicated with unfilled triangles that point up (forward strand) or down (reverse strand). The location of HPRs is indicated with a box, shaded according to its respective genome.
(8.50 MB EPS)

Figure S2 Cumulative nucleotide skews along the Dhc strains VS and BAV1 genomes and circular map of strain BAV1. (Top) Shading indicates the specific skew type of each line, provided in the legend. Cumulative Combined Skew (CCskew) is the linear combination of the T-A and C-G skews. The origin of replication (Ori) is at the left and right edges of each dataset because genomic coordinates begin with the predicted Ori. Terminus of replication (Ter) is predicted at the CCskew global minimum. Local (within-replichore) reversals in slope of CCskew indicate genomic inversions, which are further highlighted with grey background. (Bottom) Circular map of strain BAV1 according to the same legend as in Figure 1, except Rings 3–6 now correspond to the VS, CBDB1, 195 and BAV1 genomes, respectively. A reversed order of certain orthologs predicted by CCskew is evident in this map as a reversed orientation of orthologs relative to BAV1 (inner or outer half-ring).
(3.08 MB EPS)

Figure S3 Unrooted phylogram of Dhc strains based on whole genome or 16S rRNA gene data. (Top) Phylogram of the estimated strain-level phylogenomic relationship based on nucleotide sequence of core protein-encoding genes. Core genes were ordered, oriented, and concatenated into a single core sequence for each strain. A multiple alignment of these four concatenated sequences was used to construct the Neighbor-Joining tree. (Bottom) Phylogram based on the 16S rRNA gene from isolated, enriched, as well as environmental Dhc strains. Three previously described phylogenetic subgroups [26] are shaded in blue, red, and green to indicate the Cornell, Victoria and Pinellas subgroups, respectively. These Dhc 16S rRNA genes are all ≥99% identical.
(0.88 MB EPS)

Figure S4 Similarity distribution of core genes with strain BAV1 as reference. Among core genes only, aa% identity relative to BAV1 is plotted for strains VS and 195. Each point is shaded according to a continuous color map based on the location of the core gene in the genome of strain BAV1, as indicated in the color legend (top-left). Nearly all core genes in strains VS and BAV1 are comparably similar to strain BAV1, except for a small co-localized group of genes (rounded box) that are all >98% identical between strains 195 and BAV1. These genes are part of an orthologous region containing the tryptophan synthesis operon (*trp*) that is located from the start of HPR2 to *ssrA* in all four strains (loci DET1465-1506, DehaBAV1_1266-1296, cbdb_A1437-1479, DhcVS1243-1280, see *trp* in Figure 2). This entire region is unusually similar between strains 195 and the Pinellas strains (See Table S1 for coordinates used in alignment). (Inset) A phylogram based on a multiple alignment of this region in the four strains. This phylogram is incongruent with the whole-genome phylogram displayed in Figure S3.
(0.94 MB EPS)

Figure S5 Tryptophan (W) at conserved positions in *Dehalococcoides* RdhB sequences. (Top) Alpha helix prediction and transmembrane region prediction in the consensus RdhB. The red, green, and blue lines of the ‘TM Prediction Plot’ indicate the strength with which the corresponding residue is predicted to be located at the inner membrane, transmembrane, or outermembrane, respectively. These predictions were calculated using a Transmembrane Hidden Markov Model (TMHMM version 0.7) plugin to Geneious, contributed by Mark A. Suchard [56]. Green horizontal cylinders indicate the location and prediction strength of presumed alpha helices, supporting the hypothesis that these are three distinct membrane-spanning helices. Though shown here only for the consensus sequence, this pattern of TM helices is consistently predicted for individual RdhB of *Dehalococcoides*. (Bottom) Sequence logo representing the alignment of all *Dehalococcoides* RdhB in the four genomes. Letter height represents the statistical support (in bits) for a residue at a given position in the sequence. Arrows (very top) emphasize the position of conserved tryptophan residues.
(1.40 MB EPS)

Figure S6 Three strain multiple alignment of the region surrounding a putative deletion event in Dhc *ethenogenes* 195. The deletion appears to have occurred intragenically such that the 3′ 1400 bp of *rdhA* DET1535 are orthologous to the corresponding positions of DhcVS1399/cbdb_A1595, while the 5′ 100 bp are orthologous to corresponding positions in DhcVS1427/cbdb_A1624. The chimeric nature of DET1535 is further supported by local synteny, and the apparent deletion accounts for the absence of these genes in strain 195. Genes are shaded according to the following: *rdhAB* - red, other *rdh* associated genes - blue, hypothetical genes - white, other genes for which a functional annotation was assigned - light blue. For concision, annotations are often indicated at only one gene in a vertically aligned (orthologous) group. Locus ID are shown as space permits, with preference given to genes discussed in the main text. Genomic location is labeled above the black horizontal bar of each respective genome, with a much thinner horizontal line indicating a gap. A bar plot of local nucleotide identity is shown above the annotated multiple alignment and shaded to reinforce contrast in values. Green indicates windows of near perfect identity. BAV1 is not shown because this region is absent in BAV1, occurring within a larger apparent deletion in its genome.
(0.30 MB PDF)

Figure S7 Neighbor Joining consensus tree of the deduced amino acid sequence from all full-length *rdhA* genes available from six *Dehalococcoides* (Dhc) strains as well as non-Dhc microorganisms. Genes are labeled by gene ID or accession number and shaded in red, light green, dark green, blue, grey or brown if they are derived from Dhc strains VS, BAV1, CBDB1, 195, other Dhc strains, or non-Dhc, respectively. Bootstrap values are given at the tree nodes. Thin purple, red, and grey vertical lines denote 0.01, 0.05, and 0.10 Jukes-Cantor distances, respectively, cutoff values used in the classification of orthologs, paralogs, and duplications shown in Figure 2. Previously assigned orthologous groups [10] are indicated here with the same numeric assignments and expanded to include new members where appropriate. By this nomenclature the three core *rdhA* are members of groups 10, 11, and 12. These *rdhA* form two major phylogenetic clusters. Cluster 1 includes only Dhc *rdhA* and core *rdhA* group 10. Cluster 2 includes core groups 11 and 12, as well as DhcVS1349, cbdb_A1503, cbdb_A1539, and all non-Dhc *rdhA* available in the public database. Abbreviations: cp orthochlorophenol; dcp - 3,5-dichlorophenol; dca - dichloroethane; RD - reductive dehalogenase; Desulfitob - *Desulfitobacterium*; Sulfurosp - *Sulfurospirillum*; Dehalob - *Dehalobacter*; Photobac - *Photobacterium*.
(0.60 MB EPS)

Figure S8 Approximate reconstruction of the HPRs in the most recent common ancestor (MRCA) of the four contemporary Dhc strains. The phylogenetic tree is taken from the core genome phylogeny shown in Figure S3. The block-representation of the HPRs is taken from Figure 2. Each unique color denotes an orthologous region shared between two or more strains. No-fill denotes a region unique to that strain. Black tick marks indicate the location of *rdhA* genes. A total of 19 *rdhA* are predicted in the MRCA, but it should be noted that this is the estimated maximum, excluding *rdhA* that have been lost in all four strains since divergence.
(0.78 MB EPS)

Figure S9 Vinyl chloride reductase genomic islands (GEIs) of strains VS (bottom) and BAV1 (top). A mauve and green highlight indicates the respective *bvcA* and *vcrA* island boundaries with genomic coordinates shown at top-facing tick marks. Genes are shaded according to the following: *rdhAB* - red, other *rdh* associated genes - blue, tmRNA-enoding gene, *ssrA*, and its direct repeat (DR) - purple, tRNA genes and repeated fragments - green, hypothetical genes - white, genes associated with recombination - black, other genes for which a functional annotation was assigned - light blue. A cluster of six recombination associated genes (DhcVS1282-1287) are located immediately following *ssrA* at the beginning of *vcrA*-GEI. This cluster has syntenic homologs immediately following the repeated gene fragment at the downstream boundary of *vcrA*-GEI (DhcVS1292-1298), suggesting a separate, earlier integration of another *ssrA* GEI. An additional 10 *ssrA* DRs are interspersed among rdhA downstream (right) of *vcrA*-GEI.
(0.43 MB EPS)

Table S1 Comparison of genomic features for the four *Dehalococcoides* genomes and their HPRs. Values for strains 195 [9] and CBDB1 [10] are from their respective publications. Number of shotgun Sanger reads was obtained from the Ensembl Trace Server (http://trace.ensembl.org/). Raw sequencing data was not publicly available for strain CBDB1. However, it was sequenced to a reported coverage of 12× [10], providing for an estimate of total reads with an assumed average read length of 750 bp. “Number repeated elements” refers to perfectly repeated elements greater than 18 bp, detected by an exhaustive repeat search with the repeat-match algorithm in MUMmer3 [68].
(0.24 MB PDF)

Table S2 Differences in predicted core metabolism between the four *Dehalococcoides* genomes. Each column provides the presence of a gene in the respective genome as its locus ID, or “ND” for Not Detected. A brief annotation summary is provided for each core metabolism gene group (row).
(0.22 MB PDF)
