# Supplementary figures and images for: Localized Plasticity in the Streamlined Genomes of Vinyl Chloride Respiring Dehalococcoides
Source: PLoS Genet. 2009 Nov 6;5(11):e1000714. doi: 10.1371/journal.pgen.1000714 (PMC2764846; doi:10.1371/journal.pgen.1000714)

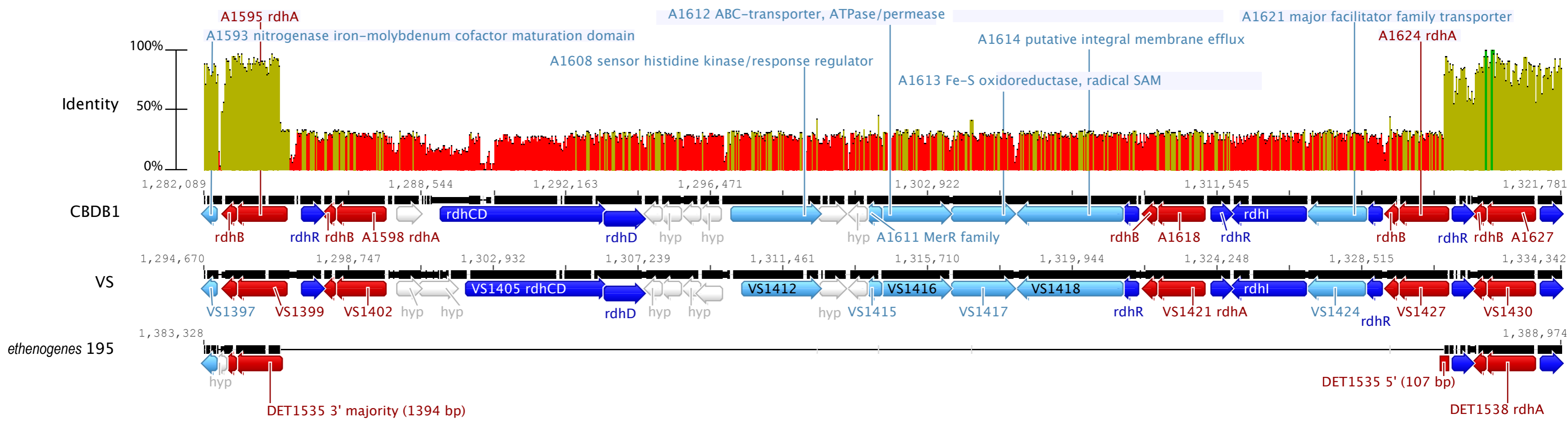

Supplement: Figure S6 — Three strain multiple alignment of the region surrounding a putative deletion event in Dhc ethenogenes 195. The deletion appears to have occurred intragenically such that the 3′ 1400 bp of rdhA DET1535 are orthologous to the corresponding positions of DhcVS1399/cbdb_A1595, while the 5′ 100 bp are orthologous to corresponding positions in DhcVS1427/cbdb_A1624. The chimeric nature of DET1535 is further supported by local synteny, and the apparent deletion accounts for the absence of these genes in strain 195. Genes are shaded according to the following: rdhAB - red, other rdh associated genes - blue, hypothetical genes - white, other genes for which a functional annotation was assigned - light blue. For concision, annotations are often indicated at only one gene in a vertically aligned (orthologous) group. Locus ID are shown as space permits, with preference given to genes discussed in the main text. Genomic location is labeled above the black horizontal bar of each respective genome, with a much thinner horizontal line indicating a gap. A bar plot of local nucleotide identity is shown above the annotated multiple alignment and shaded to reinforce contrast in values. Green indicates windows of near perfect identity. BAV1 is not shown because this region is absent in BAV1, occurring within a larger apparent deletion in its genome. (0.30 MB PDF) [file pgen.1000714.s006.pdf]
