# Supplementary material for: Localized Plasticity in the Streamlined Genomes of Vinyl Chloride Respiring Dehalococcoides
Source: PLoS Genet. 2009 Nov 6;5(11):e1000714. doi: 10.1371/journal.pgen.1000714 (PMC2764846; doi:10.1371/journal.pgen.1000714)
Supplement: Table S1 — Comparison of genomic features for the four Dehalococcoides genomes and their HPRs. Values for strains 195 [9] and CBDB1 [10] are from their respective publications. Number of shotgun Sanger reads was obtained from the Ensembl Trace Server (http://trace.ensembl.org/). Raw sequencing data was not publicly available for strain CBDB1. However, it was sequenced to a reported coverage of 12× [10], providing for an estimate of total reads with an assumed average read length of 750 bp. “Number repeated elements” refers to perfectly repeated elements greater than 18 bp, detected by an exhaustive repeat search with the repeat-match algorithm in MUMmer3 [68]. (0.24 MB PDF) [file pgen.1000714.s010.pdf]

	195		CBDB1		BAV1		VS	
genome length (bp)	1469720		1395502		1341892		1413462	
%(G+C)	48.9		47.0		47.2		47.3	
Total CDS	1580		1458		1371		1442	
% of total CDS that are core	68.31		72.63		76.65		73.37	
total *rdhA*	17		32		11		36	
position of *trp*-region of atypically high similarity	1338813 - 1362043		1137770 - 1165834		1243905 - 1267136		1150360 - 1177845	
Number repeated element per 10 kbps	5.3		10.2		4.5		6.2	
total strain-specific CDS	360		170		157		163	
Median length of intergenic spacers	34		37		37		37	
Number of Shotgun Reads	26703		23000		29941		35287	
	HPR1	HPR2	HPR1	HPR2	HPR1	HPR2	HPR1	HPR2
HPR start	57857	1325459	58100	1137418	57519	1243554	57524	1149439
HPR end	308964	1423822	231199	1349625	312176	1296003	259060	1367589
Number of strain-specific CDS in HPRs	115	37	31	60	79	9	48	63
rdhA in HPRs	9	7	7	24	9	0	6	30
Number repeated elements per 10 kbps	5.8	7.3	11.1	39.4	7.7	4.4	4.8	20.4
