# Supplementary material for: Localized Plasticity in the Streamlined Genomes of Vinyl Chloride Respiring Dehalococcoides
Source: PLoS Genet. 2009 Nov 6;5(11):e1000714. doi: 10.1371/journal.pgen.1000714 (PMC2764846; doi:10.1371/journal.pgen.1000714)
Supplement: Table S2 — Differences in predicted core metabolism between the four Dehalococcoides genomes. Each column provides the presence of a gene in the respective genome as its locus ID, or “ND” for Not Detected. A brief annotation summary is provided for each core metabolism gene group (row). (0.22 MB PDF) [file pgen.1000714.s011.pdf]

BAV1	CBDB1	DET195	VS	Annotation
ND	ND	DET_0095	ND	ferrous iron transport protein B, FeoB
ND	ND	DET_0096	ND	feoA family protein
ND	ND	DET_0097	ND	iron dependent repressor, putative
BAV1_0151	ND	DET_0202	VS190	histidinolphosphate phosphatase family protein
BAV1_0150	ND	DET_0203	VS191	glycosyl transferase family
BAV1_0149	ND	DET_0204	ND	NAD dependent epimerase/dehydratase
BAV1_0148	ND	DET_0205	ND	Nucleotidyl transferase
BAV1_0147	ND	DET_0206	ND	GHMP kinase
BAV1_0146	ND	DET_0207	ND	sugar isomerase SIS
BAV1_0145	ND	DET_0208	ND	glycosyl transferase family 2
BAV1_0144	ND	DET_0209	ND	hypothetical protein
BAV1_0143	ND	DET_0210	ND	glycosyl transferase family 2
BAV1_0142	ND	DET_0211	ND	glycosyl transferase group 1
BAV1_0140	ND	DET_0213	ND	hypothetical protein
BAV1_0139	ND	DET_0214	ND	coenzyme F420 hydrogenase/dehydrogenase beta subunit domain-like protein
BAV1_0138	ND	DET_0215	ND	polysaccharide biosynthesis protein
ND	cbdbA247	DET_0240	VS78	tetrapyrrole methylase family protein
ND	cbdbA248	DET_0241	VS76	radical SAM domain protein
ND	cbdbA249	DET_0242	VS75	conserved hypothetical protein
ND	cbdbA250	DET_0243	VS74	radical SAM domain protein
ND	cbdbA251	DET_0244	VS73	ribonucleotide reductase cobalamin-dependent, NrdJ
ND	cbdbA253	DET_0245	VS72	cob(I)alamin adenosyltransferase, CobA
ND	cbdbA254	DET_0246	VS71	cobalamin biosynthesis protein, CobD
ND	cbdbA255	DET_0247	VS70	conserved hypothetical protein
ND	cbdbA257	DET_0248	VS69	cysteine desulfurase
ND	cbdbA258	DET_0249	VS68	CbiZ-like protein
ND	cbdbA259	DET_0250	VS76	iron ABC transporter periplasmic iron binding protein, putative
BAV1_0980	cbdbA1081	DET_1147	ND	ferric uptake regulator, Fur family
ND	ND	DET_1148	ND	nitrogenase cofactor biosynthesis protein, NifB
ND	ND	DET_1149	ND	acetyltransferase GNAT family
ND	ND	DET_1150	ND	ferredoxin, [2Fe2S]
ND	ND	DET_1151	ND	dinitrogenase iron molybdenum cofactor NifB Y X family protein
ND	ND	DET_1152	ND	nitrogenase molybdenum iron protein beta subunit
ND	ND	DET_1153	ND	nitrogenase MoFe cofactor biosynthesis protein, NifE
ND	ND	DET_1154	ND	nitrogenase molybdenum iron protein, beta subunit NifK
ND	ND	DET_1155	ND	nitrogenase molybdenum iron protein, alpha chain, NifD
ND	ND	DET_1156	ND	nitrogen regulatory protein P II
ND	ND	DET_1157	ND	nitrogen regulatory protein P II
ND	ND	DET_1158	ND	nitrogenase iron protein, NifH
ND	ND	DET_1159	ND	molybdenum ABC transporter ATP binding protein
ND	ND	DET_1160	ND	molybdenum ABC transporter permease protein modB
ND	ND	DET_1161	ND	molybdenum ABC transporter periplasmic molybdate binding protein, ModA
ND	ND	DET_1162	ND	transcriptional regulator, putative
ND	ND	DET_1174	ND	Fec type ABC transporter periplasmic iron binding protein
ND	ND	DET_1175	ND	Fec type ABC transporter permease protein
ND	ND	DET_1176	ND	Fec type ABC transporter ATP binding protein
ND	ND	DET_1178	ND	transcriptional regulator MarR family
BAV1_1291	cbdbA1472	DET_1501	ND	flavodoxin
ND	cbdbA1593	DET_1532	VS1397	dinitrogenase iron molybdenum cofactor family protein
BAV1_0218	cbdbA181	ND	VS161	L-threonine aldolase
BAV1_0238	cbdbA155	ND	VS140	L-alanine dehydrogenase
BAV1_0663	cbdbB17	ND	ND	oxidoreductase, molybdopterin binding
BAV1_0939	cbdbA1034	ND	VS928	ABC transporter, ATPase subunit
BAV1_0940	cbdbA1036	ND	VS929	ABC transporter, ATPase subunit
BAV1_0941	cbdbA1037	ND	VS930	ABC transporter, cytoplasmic membrane component
BAV1_0942	cbdbA1038	ND	VS931	Nickel transporting ATPase
BAV1_0943	cbdbA1039	ND	VS932	extracellular solute-binding protein family 5
BAV1_1054	cbdbA1160	ND	VS1026	arsenical resistance protein
BAV1_1328	cbdbA1667	ND	VS1458	NAD dependent epimerase/dehydratase
BAV1_1329	cbdbA1668	ND	VS1459	NAD dependent epimerase dehydratase
ND	cbdbA1568	ND	VS1369	glutamate-ammonia ligase
